# Fetal neonatal alloimmune thrombocytopenia treatment with intravenous immunoglobulin: a challenge in pregnancy management and infection assessment ‒ case report

**DOI:** 10.1515/crpm-2021-0095

**Published:** 2022-02-01

**Authors:** Sara Bernardes da Cunha, Maria Carolina Fortuna Carneiro, Inês Falcão Reis, Cátia Rasteiro, Augusta Pinto, Teresa Paula Teles

**Affiliations:** Gynecology and Obstetrics Department, Centro Hospitalar de Entre o Douro e Vouga, Santa Maria da Feira, Portugal; Universidade da Beira Interior, Covilhã, Portugal

**Keywords:** fetal neonatal alloimmune thrombocytopenia, immunoglobulin treatment, pregnancy surveillance

## Abstract

**Objectives:**

Fetal and neonatal alloimmune thrombocytopenia is a rare condition associated with fetal and neonatal morbimortality. Prevention of recurrence includes intravenous immunoglobulin. One challenge in pregnancy surveillance remains the fact that maternal intravenous immunoglobulins therapy can result in false-positive infectious markers. The goal of this case report is to highlight the possible serological misdiagnosed infection associated with intravenous immunoglobulins therapy in pregnancy, and the difficulty of management in this time of a women’s life.

**Case presentation:**

We report a case of a 38-year-old pregnant woman, with a previous affected child with fetal neonatal alloimmune thrombocytopenia. To prevent recurrence, intravenous immunoglobulin treatment was administered in early second trimester. In the second trimester routine analysis, a positive anti-treponemal test and a toxoplasmosis seroconversion occurred. Infection suspicion based on test positivity of some infectious agents, after passive acquired antibodies, can lead to anxiety and subsequent unnecessary treatment.

**Conclusions:**

Clinicians and pathologists must be aware of the possible acquisition of these antibodies during treatment and be able to counsel patients receiving intravenous immunoglobulin. Managing possible infectious intercurrences in pregnancy remains a challenge.

## Introduction

Fetal and neonatal alloimmune thrombocytopenia (FNAIT) is a rare condition associated with fetal and neonatal morbimortality [1]. It is considered the most common cause of fetal and neonatal severe thrombocytopenia [2], [3], [4] with 1/1,000 incidence in pregnancy [2, 4, 5].

All platelets have natural proteins on their surface called human platelet antigens (HPAs). In FNAIT, fetal platelets circulating in the mothers bloodstream cause a immunological reaction due to exposition to a different platelet antigen resulting in antibodies formation [1, 6, 7]. The most commonly implicated antibody is anti-HPA-1a (80%) [1, 2, 8]. The IgG antibodies cross the placenta and bind to platelets in fetal circulation signalizing them to be removed by reticuloendothelial system [2]. This can cause platelet destruction and inhibits fetal platelet formation leading to hemostasis deficiency and hemorrhagic complications [9]. Vascular integrity and angiogenesis are also affected [8].

Clinical manifestations range from asymptomatic (mild to moderate thrombocytopenia) to more severe manifestations. Intracranial hemorrhage (ICH) occurs in 7–26% of untreated children, 80% of which antenatally, traducing a mortality of 10% and neurological consequences in 20% [7, 10], [11].

Antenatal treatment aims to prevent ICH. However, an optimal approach has yet to reach consensus [7]. The treatment efficacy is based in case series and a few randomized trials. Treatments can include fetal intrauterine platelet transfusions (IUPT) in case of thrombocytopenia after fetal blood sampling (FBS), and/or weekly maternal high dose intravenous immunoglobulins (IVIG) and/or maternal corticosteroids [7].

IVIG is derived from the plasma [12]. When under the use of IVIG during pregnancy, a new challenge presents, as maternal IVIG therapy could result in false-positive infectious markers. This can prevent the mother from donating her own platelets and can also lead to infectious suspicion and subsequent inadvertent treatment. Previous testing can allow an easier identification of falsely positive infectious markers [4]. Also, *a posteriori* testing can help to ascertain that the results were undoubtedly false positive, since passively acquired antibodies from IVIG administration generally disappear within 4 months after administration of the final dose [12].

Regarding the management of this particular situation during pregnancy, quality evidence is still lacking. We describe a clinical case where the immunoglobulin treatment can mislead the diagnosis of infectious disease of pregnancy, some of these with potential fetal and maternal complications. The purpose of this case report is to highlight the potential issues regarding infectious screening in FNAIT under preventive treatment with IVIG.

## Case presentation

A 38-year-old, O negative, pregnant woman in her fourth gestation was referred to our hospital for pregnancy surveillance. A first trimester spontaneous abortion resulted from the first gestation; no specific exams were conducted. The second pregnancy fetal demise was diagnosed at 16 weeks of pregnancy. Only a placental intervillositis was found on laboratory workup study.

On the third pregnancy, serological studies and sonographic evaluation were normal until 34 weeks of gestation. In the third trimester ultrasound, a left ventriculomegaly (VMG) (10 mm) was detected. A live newborn was born by vaginal delivery at term, with an Apgar score 9/10/10 at first, second and third minutes respectively and neonatal weight of 2,830 g. After delivery, physical exam revealed petechiae spread across the chest, left flank, and abdomen. A severe thrombocytopenia (32 × 10^9^/L) was diagnosed requiring platelet transfusion. The neurological examination was normal, however transfontanellar ultrasound confirmed the VMG previously diagnosed, measuring 10 mm, without any sign of ICH. No further imagological studies were conducted. The placenta anatomopathological report identified mild chronic intervillositis.

Neonatal thrombocytopenia etiology assessment was performed. Although maternal platelet count was normal, the maternal and paternal platelet antigen serological typing revealed that the father was homozygotic for HPA-1a and the mother was homozygotic for HPA-1b. Moreover, maternal serum was positive for HPA antibodies. In respect to HLA genotypes father was homozygotic HPA-1a and mother was homozygotic to HPA-1b and maternal serum was positive for HPA-1a antibodies. Thus, the diagnosis of FNAIT was established. At four years old by the time of the mothers fourth pregnancy, the only known clinical impairment was VI nerve palsy.

On the fourth pregnancy, high dose IGIV (1 g/kg/week) was started at 14 weeks of gestation and prednisolone (0.5 mg/kg/day) at 20 weeks. The dose administered remained stable throughout the pregnancy. In the second trimester routine serologic evaluation, a positive anti-treponemal test and a toxoplasmosis seroconversion were identified. Previous syphilis screening was assessed with a VDRL test, with a negative result. In the second trimester, after positive treponemal test, reflex non treponemal testing (VDRL) was negative. The patient was previously not immune to toxoplasmosis but by second trimester a positive IgG with negative IgM were offered in the routine evaluation. Time lapse between first and second trimester analysis was 15 weeks. After debating all possibilities, and taking into account the patient wishes, treatment of a possible late latent syphilis and prevention of fetal toxoplasmosis was initiated. Remaining pregnancy was uneventful with no recorded signs of fetal affection in ultrasound examinations.

A cesarean delivery for breech presentation was decided at 37 weeks of pregnancy. The neonate was born with 2,660 g and an Apgar score of 9/10/10 at first, second and third minutes respectively. Newborn platelet count from umbilical cord was 262 × 10^9^/L and physical examination was unremarkable. Currently with 12 months, the child has normal neurodevelopment. Reassessment of maternal serologies confirmed toxoplasmosis IgG and anti-treponemal negativity, six months after delivery.

## Discussion

In this case, we report a subsequent pregnancy after a FNAIT diagnosis. The estimated rate of recurrence can be as high as 79% for severe bleeding complications [11].

According to literature, several strategies can be used in the management of FNAIT. Nowadays, platelet transfusion takes a second place in FNAIT treatment since it is associated with fetal complications reported up to 11% in some studies [9]. The most useful predictor of disease severity is ICH in previous sibling, which hasn’t occurred in this case [1]. However, based in the first neonate outcome, the pregnancy risk was high, since the first newborn had VMG and VI nerve palsy was associated to FNAIT.

IVIG derived from donor plasma is used in the treatment of a variety of disorders. Children exposed to IVIG or consecutive blood transfusion have shown normal neurological, immunological and physical development. Potential risks for the mother include renal dysfunction, hemolytic anemia, aseptic meningitis, thrombotic complications, transmission of bloodborne diseases, headache and fever [2, 13, 14]. The absence of treatment, however, can result in worse developmental and behavioral outcomes [15].

Some clinically important laboratory abnormalities are described and can lead to misleading conclusions. Serologic tests, antinuclear antibodies, antinuclear cytoplasmic antibodies and rheumatoid factor may become falsely positive [16], [17], [18], but data is scarce. Moreover, the risk of a potential maternal-fetal infection is relevant and serological studies are a significant part of pregnancy surveillance.

In addressing this type of case, it is essential to distinguish a recent infection or a passive immunoglobulin side effect.

Reaginic tests can be helpful for syphilis infection diagnosis. Non-treponemal tests, sometimes used in low-risk pregnancy screening, are non-specific and can be falsely positive. A positive non-treponemal test should be confirmed with a specific treponemal test. However, a treponemal test can remain positive even after treatment [14, 19]. Neonatal false-positive test can occur due to maternal immunoglobulin treatment [19].

For the approach of toxoplasmosis infection, it is essential the knowledge of previous infection and the current determination of immunoglobulin (IgG and IgM). Performing avidity tests can help in determining the time of infection [18].

The presence of symptoms associated with infection or history of exposure can also guide the pregnancy surveillance conduct. The infections incidence in the population should also be taken into account. Ultrasound evaluation is also important in the management of pregnancies submitted to IVIG therapy. In presence of fetal infections ultrasound findings as ventriculomegaly, intracranial bleeding, intracranial calcifications, microcephaly, ascites, hepatosplenomegaly and hydrops [20].

Amniocentesis could be performed in case of high grade of suspicion and ultrasound abnormalities, however, it is associated with a risk of fetal loss that should be taken into consideration and can be associated with false negative results [20].

Since there is a risk of infection, a diagnosis far more difficult in a pregnant woman treated with IVIG, these questions should be discussed with the patient. A multidisciplinary management of this cases is fundamental [14, 19].

## Conclusions

IVIG therapy could result in false-positive infectious markers misleading an erroneous diagnosis during pregnancy. The recognition of this condition allows a better interpretation of pregnancy exams and could permit a more accurate information of infectious status preventing ambiguous information to the couple and unnecessary therapeutic. It is important for clinicians to be aware of the potential for false-positive antibody results in patients who have been given immunoglobulin. The growing evidence resulting from clinical reports like ours, can help in the management of pregnant women submitted to IGIV treatment and contribute to the elaboration of guidelines for such situations.
